# Combined Targeting of PD-1 and TIM-3 in Patients with Locally Advanced or Metastatic Non–Small Cell Lung Cancer: AMBER Part 2B

**DOI:** 10.1158/1078-0432.CCR-25-0806

**Published:** 2025-06-24

**Authors:** Diwakar Davar, Zeynep Eroglu, Mohammed Milhem, Carlos Becerra, Martin Gutierrez, Antoni Ribas, Brian Di Pace, Tianli Wang, Hailei Zhang, Srimoyee Ghosh, Niranjan Yanamandra, Theo Borgovan, Shyam Srivats, Angela Waszak, Arindam Dhar, Patricia LoRusso

**Affiliations:** 1 Department of Medicine - Hematology/Oncology, Hillman Cancer Center, University of Pittsburgh Medical Center, Pittsburgh, Pennsylvania.; 2 Department of Cutaneous Oncology, Moffitt Cancer Center, Tampa, Florida.; 3 Department of Internal Medicine, University of Iowa, Iowa City, Iowa.; 4 The US Oncology Network, Dallas, Texas.; 5 Department of Oncology, Hackensack Meridian Health, Hackensack, New Jersey.; 6 Jonsson Comprehensive Cancer Center, University of California Los Angeles, Los Angeles, California.; 7 GSK, Collegeville, Pennsylvania.; 8 GSK, Waltham, Massachusetts.; 9 Yale University, New Haven, Connecticut.

## Abstract

**Purpose:**

Treatment options for patients with non–small cell lung cancer (NSCLC) who have progressed on anti–PD-(L)1 treatment are lacking. In preclinical models, blockade of the inhibitory immune receptor T-cell immunoglobulin and mucin-domain containing protein 3 (TIM-3) is associated with an antitumor response. AMBER (NCT02817633) part 2B assessed the safety and efficacy of cobolimab, an anti–TIM-3 humanized monoclonal antibody, plus PD-1 inhibitor dostarlimab in patients with locally advanced or metastatic NSCLC who had progressed on anti–PD-(L)1 treatment.

**Patients and Methods:**

Adult patients with anti–PD-(L)-1 treated locally advanced or metastatic NSCLC were included. Patients received cobolimab 100, 300, or 900 mg and dostarlimab 500 mg every 3 weeks. Treatment continued until disease progression, unacceptable toxicity, patient withdrawal, investigator’s decision, or death. Endpoints included overall response rate, disease control rate, and safety. *Post hoc* biomarker assessments of pretreatment tumor biopsies were also assessed.

**Results:**

In total, 85 patients were enrolled and 84 received treatment. Treatment-emergent and treatment-related adverse events occurred in 98.8% and 52.4% of patients, respectively; no treatment-related deaths occurred. Across all three cobolimab doses, overall response rate was 8.3% (95% confidence interval, 3.4–16.4) and disease control rate was 21.4% (95% confidence interval, 13.2–31.7); both were highest in the 300 mg cohort (*n* = 41; 9.8% and 22.0%). Pretreatment TIM-3 expression was significantly greater in patients with partial or stable responses versus progressive disease.

**Conclusions:**

Cobolimab plus dostarlimab showed preliminary efficacy and tolerability in a subset of patients with locally advanced/metastatic NSCLC.

*See related article by Davar et al., p. 3433*

**Translational Relevance**

AMBER part 2B assessed cobolimab, a T-cell immunoglobulin and mucin-domain containing protein 3 mAb, in combination with the PD-1 mAb dostarlimab in patients with locally advanced/metastatic non–small cell lung cancer who have progressed on prior anti–PD-(L)1 treatment. The treatment combination was well tolerated with early evidence of efficacy for a subset of patients. These findings support the continued investigation of combined dual checkpoint inhibition with cobolimab plus dostarlimab in PD-(L)1–relapsed/refractory solid tumors.

## Introduction

Programmed cell death protein 1 (PD-1) is a receptor expressed by activated T and B cells which binds to PD-L1, also known as B7-H1 (1, 2), and PD-L2, also known as B7-DC (3, 4), resulting in suppression of cytotoxic functions. Anti–PD-(L)1 mAbs are immune checkpoint inhibitors (ICI) that disrupt the PD-1/PD-L1 interaction, thus augmenting T-cell function and antitumor activity (5–7). Consequently, ICIs have improved progression-free survival (PFS) and overall survival (OS) in patients with advanced cancers including melanoma, renal cell carcinoma, and non–small cell lung cancer (NSCLC) in pivotal phase III studies (8–16).

Lung cancer is associated with a poor prognosis worldwide and is the leading contributor to cancer-related mortality (17), with NSCLC accounting for approximately 81% of lung cancer cases in the United States (18). Patients without a driver genomic aberration [e.g., epidermal growth factor receptor (*EGFR*), anaplastic lymphoma kinase (ALK), c-ROS oncogene 1 (*ROS1*), v-raf murine sarcoma viral oncogene homolog B1 (*BRAF*), etc.] typically receive anti–PD-(L)1 antibodies as a monotherapy or in combination with platinum-based chemotherapy (19). In the PERLA study, the addition of dostarlimab, a humanized anti–PD-1 mAb, that binds the PD-1 receptor in platinum-based chemotherapy in previously untreated patients with advanced non–squamous NSCLC without known targetable genomic aberrations demonstrated comparable survival outcomes in patients who received pembrolizumab and platinum-based chemotherapy (20), supporting further investigation of dostarlimab in combination with other treatments in NSCLC.

T-cell immunoglobulin and mucin-domain containing protein 3 (TIM-3) is an inhibitory immune receptor that is widely expressed on multiple immune cells, including T cells, natural killer (NK) cells, monocytes, and dendritic cells, and is implicated in the regulation of both adaptive and innate immune responses (21, 22). Evidence in preclinical models of cancer has indicated that TIM-3 blockade augments antitumor immunity through effects on both T cells and dendritic cells (23–25). In patients with NSCLC, high TIM-3 expression on tumor-infiltrating lymphocytes and myeloid-derived cells has been associated with a poor prognosis (26–28). Further, in NSCLC explant models, although TIM-3 blockade alone had a limited effect on tumor growth, the combined blockade of TIM-3 and PD-1 augmented immune function and resulted in antitumor responses (21, 29, 30).

Cobolimab (TSR-022/GSK4069889) is a high-affinity anti–TIM-3 humanized mAb which has been shown to promote T-cell activity in *ex vivo* T-cell stimulation assays (31). Cobolimab is being assessed in an ongoing, two-part (dose escalation and expansion), multicenter, open-label, phase I multicohort study (AMBER, NCT02817633). This study is assessing cobolimab as monotherapy and in combination with other drugs in advanced solid tumors. In part 1 of AMBER, cobolimab was shown to be well tolerated as a monotherapy (part 1a) and in combination with dostarlimab (part 1c) for patients with advanced solid tumors, including NSCLC (32). Here, the results from AMBER part 2B are reported, which evaluated the safety and efficacy of cobolimab in combination with dostarlimab in patients with locally advanced or metastatic NSCLC whose disease had progressed on prior anti–PD-(L)1 treatment.

## Patients and Methods

### Study design and conduct

AMBER (NCT02817633) is a multicenter, open-label, phase I study assessing cobolimab in combination with dostarlimab, with part 2B assessing patients with locally advanced or metastatic NSCLC who had documented disease progression on prior anti–PD-(L)1 treatment (Supplementary Fig. S1). The study start date was July 2016, and the data cutoff for the analyses presented here was February 2023.

The study was conducted in accordance with ethical principles founded in the Declaration of Helsinki in accordance with the protocol. The study protocol, amendments, informed consent, and all other information that required preapproval were reviewed and approved by national, regional, or investigational center ethics committee or institutional review board, in accordance with the International Committee on Harmonization of Good Clinical Practice and applicable country-specific requirements, including US 21 CFR 312.3(b) for constitution of independent ethics committees.

### Eligibility

Full inclusion and exclusion criteria are provided in the Supplementary Materials. In brief, patients were eligible to be included if they were least 18 years of age, had an Eastern Cooperative Oncology Group (ECOG) performance status of 0 or 1, and had histologically proven locally advanced (unresectable) or metastatic NSCLC that was measurable by CT or MRI per Response Evaluation Criteria in Solid Tumours (RECIST) v1.1 (33). Patients had also received prior treatment with an anti–PD-(L)1 antibody with documented progression or had prior receipt of an EGFR tyrosine kinase or ALK inhibitor (if known *EGFR* mutation or *ALK* translocation, respectively). Ineligible patients included those who received prior treatment with anti–PD-1, anti–PD-(L)1, or anti–PD-(L)2 agents that resulted in permanent discontinuation due to an adverse event (AE) or had received prior treatment with an anti–lymphocyte-activation gene 3 or anti–TIM-3 agent.

### Treatments

Following a 21-day screening period, patients received 100, 300, or 900 mg cobolimab intravenously and 500 mg dostarlimab intravenously every 3 weeks, for approximately 2 years until disease progression, unacceptable toxicity, patient withdrawal, investigator’s decision, or death.

### Study endpoints for part 2B

The primary endpoint was a confirmed overall response rate (ORR), defined as the proportion of patients who achieved a complete response (CR) or partial response (PR), as assessed by the investigator per RECIST v1.1. Secondary endpoints included disease control rate (DCR), defined as the percentage of patients who achieved CR, PR, or standard disease (SD) for a minimum of 16 weeks, as assessed by the investigator per RECIST v1.1; immune-related (ir)ORR and irDCR as assessed by the investigator per irRECIST; PFS, defined as the time from the first dose to the earlier date of assessment of progression or death by any cause in the absence of progression by RECIST v1.1, as assessed by the investigator; and OS, defined as the time from the date of the first dose of study treatment to the date of death by any cause.

Changes to patient target lesion size from baseline were assessed. Target lesion size was calculated as the sum of longest (nonnodal) dimension and shortest (nodal) axes for all target lesions. Patients with target lesion size not available after baseline were excluded from this analysis.

### *Post hoc* analyses

Biomarker assessments of pretreatment tumor biopsies were assessed and included PD-L1 expression (tumor proportion score; Dako, 22C3 pharmDx) and TIM-3 expression (research assay using clone D5D5R), both evaluated via immunohistochemistry (IHC). TIM-3 was scored by a pathologist annotating the central tumor region in individual hematoxylin and eosin pathology images, followed by automatic random subsampling and detection of TIM-3 by automated image analysis.

Patients’ prior ICI treatment and current exposure to study treatment were also assessed, including measures of treatment duration, confirmed best overall response, and date of progression, based on patient medical records.

### Assessments and safety

CT or MRI evaluations were used to assess the extent of the disease and were conducted every 8 weeks (56 ± 7 days) from the date of the first dose of study treatment. Patients who remained on treatment after 1 year had imaging performed every 12 weeks (84 ± 7 days). Tumor assessments (radiographic and tumor markers) were performed independent of dose delays and/or interruptions and/or at any time when progression of disease was suspected. Following disease progression or treatment discontinuation, patients had safety follow-up visits 30 (±7) and 90 (±14) days after the last dose of study treatment.

For safety assessments, AEs were coded using the Medical Dictionary for Regulatory Activities (MedDRA) v25.1 and assessed by investigators according to the Common Terminology Criteria for Adverse Events (CTCAE) v4.03. AEs included treatment-emergent AEs (TEAE), treatment-related AEs (TRAE), and irTEAEs and were recorded by frequency and severity. All AEs and serious AEs were collected and recorded for each patient from the day of signed informed consent until 90 days after treatment or until initiation of alternative anticancer treatment.

### Statistical analysis

Efficacy and safety analyses were performed using the safety population, defined as all patients who receive any amount of cobolimab. A sample size of approximately 132 patients (up to 44 per treatment arm) was planned for part 2B in accordance with a Simon two-stage minimax design (34), with the final sample size for each dose level determined following the ongoing examination of the totality of data observed, including receptor occupancy, safety, and efficacy data. The minimax design assumed a null hypothesis of a 5% true response rate tested against a one-sided alternative when the true response rate is 15% (one-sided type I error rate: 10%; power: 80%). Categorical variables were summarized as numbers and percentages, and continuous variables were summarized as numbers, median, minimum, maximum, and first and third quartiles. For response rates, point estimates and Clopper–Pearson two-sided 95% confidence interval (CI) were calculated. Time-to-event efficacy analyses of PFS and OS were performed using Kaplan–Meier methods. CIs were estimated using the Brookmeyer–Crowley method (1982; ref. 35).

For the *post hoc* biomarker analyses, Spearman correlations were used to evaluate the association between TIM-3 and PD-L1, and the Wilcoxon rank-sum test was applied for TIM-3 levels associated with response.

### RRID resources

R Project for Statistical Computing (RRID: SCR_001905); cowplot (RRID: SCR_018081); tidyverse (RRID: SCR_019186); dplyr (RRID: SCR_016708); tidyr (RRID: SCR_017102), ggplot2 (RRID: SCR_014601); purrr (RRID: SCR_021267); knitr (RRID: SCR_018533); and BBrowserX (RRID: SCR_025984; utilized by BioTuring).

### Data availability

Please refer to https://www.gsk-studyregister.com/en/ to access GSK’s data sharing policies and, as applicable, seek anonymized subject-level data via https://vivli.org/ourmember/gsk/. To access data for other types of GSK sponsored research, for study documents without patient-level data, and for clinical studies not listed, please send an email to www.share-support@gsk.com.

## Results

### Patient demographics

In total, 85 patients were enrolled and 84 received treatment. Patient baseline demographics and characteristics are summarized in Table 1 and were similar across the three different cobolimab dose cohorts. In brief, the median patient age was 67.5 (range: 35–86) years, and the most common histologies were adenocarcinoma (*n* = 58; 69.0%) and squamous cell carcinoma (*n* = 22; 26.2%). Overall, 4 (4.8%) patients had known *EGFR* mutations, and 49 (58.3%) patients had received ≥3 lines of prior treatment. Supplementary Table S1 summarizes the representativeness of the study patients compared with the global patient population.

**Table 1.** Baseline patient demographics and clinical characteristics.

Characteristic	Cobolimab 100 mg + dostarlimab (*n* = 14)	Cobolimab 300 mg + dostarlimab (*n* = 41)	Cobolimab 900 mg + dostarlimab (*n* = 29)	Total (*N* = 84)
**Age, median (range), years**	63.0 (40–83)	69.0 (35–86)	68.0 (47–86)	67.5 (35–86)
**Sex, ** ***n*** ** (%)**				
Female	D^a^	D^a^	D^a^	34 (40.5)
Male	D^a^	D^a^	D^a^	50 (59.5)
**Race, ** ***n*** ** (%)**				
White	D^a^	D^a^	D^a^	73 (86.9)
De-identified	D^a^	D^a^	D^a^	11 (13.1)
**Ethnicity, ** ***n*** ** (%)**				
Not Hispanic or Latino	12 (85.7)	34 (82.9)	25 (86.2)	71 (84.5)
De-identified	D^a^	D^a^	D^a^	13 (15.5)
**ECOG performance status, ** ***n*** ** (%)**				
0	4 (28.6)	6 (14.6)	6 (20.7)	16 (19.0)
1	10 (71.4)	35 (85.4)	23 (79.3)	68 (81.0)
**Histology, ** ***n*** ** (%)**				
Adenocarcinoma	9 (64.3)	29 (70.7)	20 (69.0)	58 (69.0)
Adenocarcinoma with squamous features	1 (7.1)	0	0	1 (1.2)
Large cell neuroendocrine carcinoma	0	0	1 (3.4)	1 (1.2)
Metastatic sarcomatoid carcinoma	0	0	1 (3.4)	1 (1.2)
Sarcomatoid carcinoma	0	0	1 (3.4)	1 (1.2)
Squamous cell carcinoma	4 (28.6)	12 (29.3)	6 (20.7)	22 (26.2)
**Prior lines of treatment** ^b^ **, ** ***n*** ** (%)**				
1	0	5 (12.2)	4 (13.8)	9 (10.7)
2	5 (35.7)	13 (31.7)	8 (27.6)	26 (31.0)
3	2 (14.3)	5 (12.2)	8 (27.6)	15 (17.9)
4	2 (14.3)	8 (19.5)	4 (13.8)	14 (16.7)
≥5	5 (35.7)	10 (24.4)	5 (17.2)	20 (23.8)
**PD-** **L1 status** ^c^ **, ** ***n*** ** (%)**				
TPS ≥50%	3 (21.4)	9 (22.0)	5 (17.2)	17 (20.2)
TPS 1%–49%	3 (21.4)	6 (14.6)	9 (31.0)	18 (21.4)
TPS <1%	4 (28.6)	11 (26.8)	12 (41.4)	27 (32.1)
Missing	4 (28.6)	15 (36.6)	3 (10.3)	22 (26.2)
**Known genetic abnormality, ** ***n*** ** (%)**				
*APC*	0	1 (2.4)	0	1 (1.2)
*BRAF—*other	0	2 (4.9)	0	2 (2.4)
*EGFR*—exon 19 deletion	0	1 (2.4)	0	1 (1.2)
*EGFR*—other	1 (7.1)	1 (2.4)	0	2 (2.4)
*EGFR*—T790M	0	0	1 (3.4)	1 (1.2)
*KRAS*	4 (28.6)	4 (9.8)	5 (17.2)	13 (15.5)
*PI3K*	0	1 (2.4)	0	1 (1.2)
*TP53*	0	2 (4.9)	0	2 (2.4)
Other	3 (21.4)	7 (17.1)	7 (24.1)	17 (20.2)
Missing	6 (42.9)	22 (53.7)	16 (55.2)	44 (52.4)

Abbreviations: *APC*, adenomatous polyposis coli; *BRAF*, v-raf murine sarcoma viral oncogene homolog B1; D, deidentified; ECOG, Eastern Cooperative Oncology Group; *EGFR*, epidermal growth factor receptor; *KRAS*, Kirsten rat sarcoma viral oncogene homolog; PD-L1, programmed death ligand 1; *PI3K*, phosphoinositide 3-kinase; *TP53*, tumor protein 53; TPS, tumor proportion score.

a Data deidentified for demographic variables if at least one treatment arm had an *n* < 11.

b Main prior treatments (>50% of the total population) included carboplatin, nivolumab, and pemetrexed.

c Tested locally.

### Safety

TEAEs are summarized in Table 2. In total, TEAEs occurred in 83 (98.8%) patients, and TRAEs occurred in 44 (52.4%) patients. The incidence of TEAEs and TRAEs was similar across cobolimab doses.

**Table 2.** Overview of safety results.

AE, *n* (%)	Cobolimab 100 mg + dostarlimab (*n* = 14)	Cobolimab 300 mg + dostarlimab (*n* = 41)	Cobolimab 900 mg + dostarlimab (*n* = 29)	Total/safety population (*N* = 84)
**TEAEs**	14 (100.0)	41 (100.0)	28 (96.6)	83 (98.8)
**TRAEs**	6 (42.9)	24 (58.5)	14 (48.3)	44 (52.4)
**Grade ** **≥3 ** **TEAEs**	7 (50.0)	23 (56.1)	16 (55.2)	46 (54.8)
**Grade ** **≥3 ** **TRAEs**	2 (14.3)	5 (12.2)	4 (13.8)	11 (13.1)
**TESAEs**	6 (42.9)	17 (41.5)	15 (51.7)	38 (45.2)
**Most common TESAEs** ^a^				
Pneumonia	1 (7.1)	3 (7.3)	3 (10.3)	7 (8.3)
Back pain	0	2 (4.9)	2 (6.9)	4 (4.8)
Atrial fibrillation	1 (7.1)	2 (4.9)	0	3 (3.6)
Dyspnea	0	2 (4.9)	1 (3.4)	3 (3.6)
Hypoxia	0	1 (2.4)	2 (6.9)	3 (3.6)
Pleural effusion	0	1 (2.4)	2 (6.9)	3 (3.6)
Respiratory failure	1 (7.1)	1 (2.4)	1 (3.4)	3 (3.6)
**TRSAEs**	1 (7.1)	3 (7.3)	2 (6.9)	6 (7.1)
**irTEAEs**	1 (7.1)	10 (24.4)	5 (17.2)	16 (19.0)
**irTESAEs**	1 (7.1)	0	1 (3.4)	2 (2.4)
Pneumonitis	0	0	1 (3.4)	1 (1.2)
Pancreatitis	1 (7.1)	0	0	1 (1.2)
**TEAEs leading to study treatment discontinuation**	1 (7.1)	1 (2.4)	3 (10.3)	5 (6.0)
**TEAEs leading to study treatment delay**	4 (28.6)	9 (22.0)	5 (17.2)	18 (21.4)
**Fatal TEAEs**	1 (7.1)	1 (2.4)	2 (6.9)	4 (4.8)
**Fatal TRAEs**	0	0	0	0

Abbreviations: ir, immune-related; TEAE, treatment-emergent adverse event; TESAE, treatment-emergent serious adverse event; TRAE, treatment-related adverse event; TRSAE, treatment-related serious adverse event.

a Occurring in ≥3% of the total population.

Grade ≥3 TRAEs were experienced by 11 (13.1%) patients, and there were no deaths due to TRAEs. Thirty-eight (45.2%) patients experienced treatment-emergent serious adverse events (TESAEs), the most common of which (occurring in ≥3% of patients) were pneumonia (8.3%), back pain (4.8%), atrial fibrillation (3.6%), dyspnea (3.6%), hypoxia (3.6%), pleural effusion (3.6%), and respiratory failure (3.6%).

In total, irTEAEs were reported in 16 (19.0%) patients. Immune-related treatment-emergent serious AEs were observed for 2 (2.4%) patients and included pneumonitis (1.2%) and pancreatitis (1.2%; Table 2).

### Efficacy

Response data assessed using RECIST v1.1 and irRECIST are summarized in Table 3. Across all three cobolimab dose levels, the ORR was 8.3% (95% CI, 3.4–16.4), and the DCR was 21.4% (95% CI, 13.2–31.7). The ORR ranged from 6.9% to 9.8%, and the DCR ranged from 20.7% to 22.0% across the three cobolimab dose levels, with the highest ORR (9.8%; 95% CI, 2.7–23.1) and DCR (22.0%; 95% CI, 10.6–37.6) observed in the cobolimab 300 mg cohort (*n* = 41). The overall irORR was 9.5% (95% CI, 4.2–17.9), and the irDCR was 25.0% (95% CI, 16.2–35.6).

**Table 3.** ORR, DCR, and BOR in patients treated with cobolimab 100, 300, or 900 mg plus dostarlimab.

Response, *n* (%)	Cobolimab 100 mg + dostarlimab (*n* = 14)	Cobolimab 300 mg + dostarlimab (*n* = 41)	Cobolimab 900 mg + dostarlimab (*n* = 29)	Total/safety population (*N* = 84)
**ORR**	1 (7.1)	4 (9.8)	2 (6.9)	7 (8.3)
(95% CI)	(0.2–33.9)	(2.7–23.1)	(0.8–22.8)	(3.4–16.4)
**DCR**	3 (21.4)	9 (22.0)	6 (20.7)	18 (21.4)
(95% CI)	(4.7–50.8)	(10.6–37.6)	(8.0–39.7)	(13.2–31.7)
**BOR per RECIST v1.1**				
CR	0	0	0	0
PR	1 (7.1)	4 (9.8)	2 (6.9)	7 (8.3)
SD^a^	2 (14.3)	5 (12.2)	4 (13.8)	11 (13.1)
Progressive disease	8 (57.1)	23 (56.1)^b^	15 (51.7)	46 (54.8)
Not evaluable	1 (7.1)	3 (7.3)	2 (6.9)	6 (7.1)
Not done^c^	2 (14.3)	6 (14.6)	6 (20.7)	14 (16.7)
**irORR**	1 (7.1)	5 (12.2)	2 (6.9)	8 (9.5)
(95% CI)	(0.2–33.9)	(4.1–26.2)	(0.8–22.8)	(4.2–17.9)
**irDCR**	3 (21.4)	11 (26.8)	7 (24.1)	21 (25.0)
(95% CI)	(4.7–50.8)	(14.2–42.9)	(10.3–43.5)	(16.2–35.6)
**BOR per irRECIST**				
irCR	0	0	0	0
irPR	1 (7.1)	5 (12.2)^b^	2 (6.9)	8 (9.5)
irSD^a^	2 (14.3)	6 (14.6)	5 (17.2)	13 (15.5)
Immune-related progressive disease	8 (57.1)	20 (48.8)	14 (48.3)	42 (50.0)
Not evaluable	1 (7.1)	4 (9.8)	2 (6.9)	7 (8.3)
Not done^c^	2 (14.3)	6 (14.6)	6 (20.7)	14 (16.7)

Abbreviations: BOR, best overall response; CI, confidence interval; CR, complete response; DCR, disease control rate; ir, immune-related; ORR, overall response rate; PR, partial response; RECIST v1.1, Response Evaluation Criteria in Solid Tumors version 1.1; SD, stable disease.

a SD/irSD confirmed with a minimum of 16-week duration.

b One patient with a BOR per RECIST v1.1 of progressive disease (BOR per irRECIST of irPR) achieved an unconfirmed CR (details provided in the Supplementary Text).

c Patients in the safety population who have no postbaseline tumor assessments, including those with clinical progression and discontinuation soon after study treatment start.

**Figure 1.**
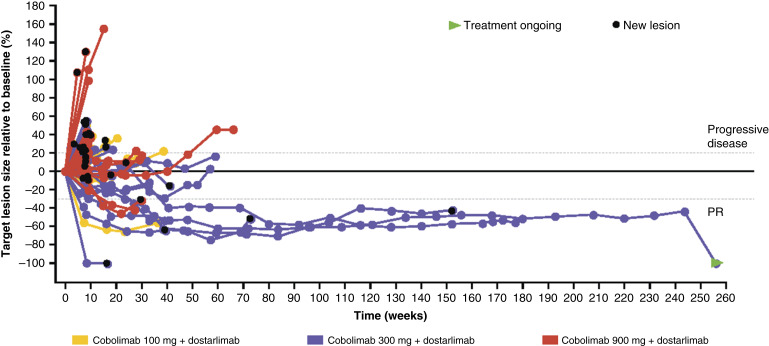
Relative change in target lesion size across cobolimab dose ranges (*N* = 69). Note: *N* number is defined as the safety population minus 15 patients with target lesion size not available after baseline, including those with no postbaseline tumor assessments.

Change in target lesion size relative to baseline is shown in Fig. 1. In responding patients, responses were often durable, including in patients who had extensive prior exposure to ICI therapy (Supplementary Fig. S2). An unconfirmed CR was observed for one patient enrolled in the cobolimab 300 mg cohort. This patient (female, ex-smoker, with *BRAF-*G446V mutation NSCLC, 65 years of age) had progressed after four prior lines of therapy (Supplementary Table S2), including *BRAF/MEK* inhibitors dabrafenib/trametinib and anti–PD-1 nivolumab (best response of SD), the latter of which the patient received 14 months prior to enrollment. Following receipt of combined cobolimab plus dostarlimab, target and nontarget lesions, including lymph nodes and adrenal lesions, were reduced, as confirmed by PET and CT imaging (Supplementary Fig. S3; Supplementary Table S3).

**Figure 2.**
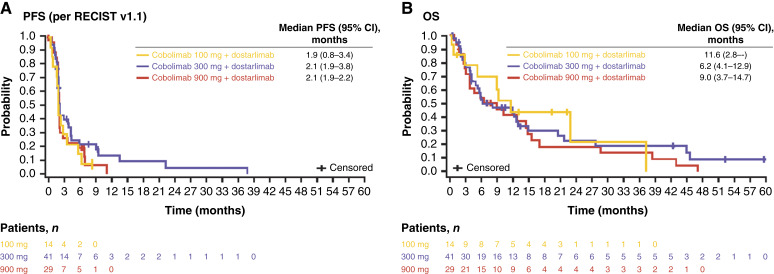
Kaplan–Meier plot of (**A**) PFS and (**B**) OS. CI, confidence interval; OS, overall survival; PFS, progression-free survival; RECIST v1.1, Response Evaluation Criteria for Solid Tumors version 1.1.

PFS and OS data for patients treated with combined cobolimab plus dostarlimab are summarized in Fig. 2. Median PFS values were similar across the three cobolimab dose cohorts. At the cobolimab 300 mg dose level, the median PFS was 2.1 months (95% CI, 1.9–3.8), and the median OS was 6.2 months (95% CI, 4.1–12.9).

### Pretreatment TIM-3 and PD-L1 expression as possible biomarkers for efficacy

**Figure 3.**
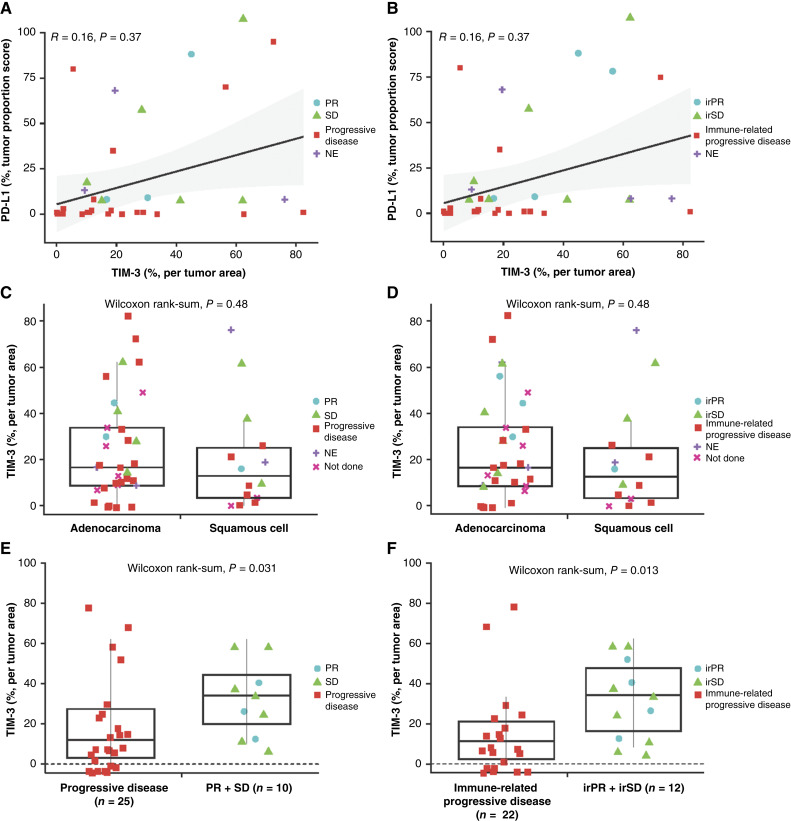
Correlation between baseline TIM-3 and PD-L1 IHC expression based on (**A**) DCR (per RECIST v1.1) and (**B**) irDCR (per irRECIST). Pretreatment TIM-3 IHC expression based on (**C**) adenocarcinoma histology, (**D**) squamous cell histology, (**E**) DCR (per RECIST v1.1)^a^, and (**F**) irDCR (per irRECIST)^b^. ^a^A response per RECIST was not evaluable for 6 patients and not done for 14 patients. ^b^A response per irRECIST was not evaluable for 7 patients and not done for 14 patients. DCR, disease control rate; ir, immune-related; NE, not evaluable; PD-L1, programmed cell death ligand 1; PR, partial response; RECIST v1.1, Response Evaluation Criteria in Solid Tumors version 1.1; SD, stable disease; TIM-3, T-cell immunoglobulin mucin domain containing protein-3, NE, not evaluable.

No correlation between baseline PD-L1 expression and TIM-3 expression was observed in patients with and without disease control, whether evaluated per RECIST v1.1 (*R* = 0.16; *P* = 0.37; Fig. 3A) or irRECIST (*R* = 0.16; *P* = 0.37; Fig. 3B). Within pretreatment tumors, TIM-3 expression was similarly distributed in patients with adenocarcinoma and squamous cell carcinoma (Fig. 3C and D); however, TIM-3 expression was significantly greater for patients with a PR or SD compared with patients with progressive disease. This result was observed whether response was evaluated by RECIST (*P* = 0.031; Fig. 3E) or irRECIST (*P* = 0.013; Fig. 3F). Conversely, pretreatment PD-L1 expression was not predictive of benefit when evaluated by histology (squamous vs. adenocarcinoma; Supplementary Figs. S4 and S5).

## Discussion

In AMBER part 2B, the safety and efficacy of the combination of cobolimab plus dostarlimab across three separate cobolimab doses was assessed in patients with locally advanced or metastatic NSCLC who had previously been treated with anti–PD-(L)1 therapy and who may have received multiple lines of therapy. Across the separate cobolimab doses, no new safety signals were observed compared with prior studies (32, 36). The observed safety profile of the treatment combination was manageable, with a low incidence of grade ≥3 TRAEs, and a toxicity profile for the combination that was consistent with those observed for single-agent dostarlimab and other anti–TIM-3/anti–PD-1 combination studies including the anti–TIM-3 treatments LY3321367 and sabatolimab (MBG453; refs. 32, 36). This finding is particularly encouraging given that 58.3% of patients assessed here had received ≥3 prior lines of treatment.

Antitumor activity was observed for combined cobolimab plus dostarlimab treatment for a number of patients across all cobolimab doses. The overall efficacy findings for the cobolimab 300 mg cohort were greater compared with responses for the cobolimab 100 and 900 mg cohorts based on RECIST and irRECIST. Taken in combination with the observed manageable safety profile and existing data on receptor occupancy, pharmacokinetics, pharmacodynamics, efficacy, and safety from other patient cohorts in the AMBER study (32, 36), the cobolimab 300 mg dose level was selected as the recommended phase II dose (RP2D). Notably, sustained responses to cobolimab plus dostarlimab treatment were observed for a number of patients, suggesting that combined cobolimab plus dostarlimab could provide durable antitumor control for select patients.

Based on the low sample sizes for phase I studies, cross-study comparisons should be considered with caution; however, despite the ORR being generally low, the observed ORR (8.3%) was favorable versus other studies evaluating anti–TIM-3 agents, including LY3321367 and sabatolimab, in patients with locally advanced tumors (37, 38). Furthermore, the patient population evaluated here was representative of real-world NSCLC—comprised of patients whose NSCLC either relapsed or was refractory to anti–PD-(L)1 and other targeted treatments (if driver genomic aberrations were present)—whereas other anti–TIM-3/anti–PD-1 combination studies have only included a subset of ICI-experienced patients (37, 38).

Although TIM-3 inhibition in other cancers has not shown favorable efficacy (39), here, TIM-3 inhibition in combination with dostarlimab showed safety and efficacy in a select number of patients. These findings align with previous preclinical data with cobolimab and dostarlimab, underscoring that PD-1/TIM-3 co-blockade was superior compared with either agent singly (40). Notably, the combination of cobolimab plus dostarlimab demonstrated an ORR of 46% in patients with PD-1–naïve hepatocellular carcinoma (41) and 43% in patients with PD-1–naïve melanoma (36).

*Post hoc* IHC analyses of TIM-3 and PD-L1 expression in pretreatment tumor samples showed TIM-3 and PD-L1 expression was similar between adenocarcinoma and squamous cell histologies; however, although PD-L1 expression was not associated with observed response, pretreatment TIM-3 levels were significantly greater for patients with an observed response than for patients with progressive disease. Given the key role of TIM-3 in regulating several immune cell types, including NK cells, myeloid cells, and effector T cells (42), identifying optimal predictive biomarkers of TIM-3 blockade will likely require deep multiomic characterization of tissue samples before determining correlations with disease severity, treatment response, and overall patient survival. Future studies are needed to further identify those patient populations most likely to benefit from TIM-3 blockade.

Key strengths of AMBER part 2B included a long follow-up period, with responses assessed up to approximately 260 weeks, and comprehensive evaluation of three dose levels of cobolimab (100, 300, and 900 mg) in combination with dostarlimab to nominate cobolimab 300 mg as the RP2D based on aggregate clinical and translational data.

The results reported here represent proof of concept for further exploration of TIM-3 blockade in patients with advanced solid tumors who had progressed on prior anti–PD-(L)1 treatment. To that end, cobolimab is currently being evaluated in combination with dostarlimab and docetaxel in COSTAR Lung (NCT04655976), an ongoing phase II/III study of patients with locally advanced or metastatic NSCLC treated with prior anti–PD-(L)1 and platinum-based doublet chemotherapies. Preclinically, docetaxel has been shown to induce the release of the alarmin high mobility group box 1 protein (HMGB1), which interacts with the TIM-3 receptor to subvert immune responses triggered by dying tumor cells (43). In addition, preclinical models have suggested that TIM-3 is associated with a promoted response to taxanes, including paclitaxel and docetaxel, via suppression of the uptake of HMGB–DNA complexes by intratumoral dendritic cells (24, 44). As such, combining docetaxel with other PD-1/TIM-3 inhibitors may provide a synergistic combination (45).

In conclusion, the combination of cobolimab plus dostarlimab showed acceptable safety and early evidence of efficacy across three cobolimab doses in a subset of patients with locally advanced or metastatic NSCLC. Based on these findings, along with previously reported receptor occupancy, pharmacokinetic, pharmacodynamic, efficacy, and safety data, cobolimab 300 mg is suggested as the RP2D. Evidence from AMBER part 2B supports continued investigation of cobolimab plus dostarlimab in this patient population.

## Supplementary Material

Supplementary Methods S1 Additional inclusion/exclusion criteria

Supplementary Figure S1 AMBER Part 2B design

Supplementary Figure S2 Post hoc analysis of prior ICI treatment and current exposure on study treatment

Supplementary Figure S3 Representative positron emission tomography (PET) and computed tomography (CT) images for the patient with an unconfirmed complete response

Supplementary Figure S4 Pretreatment TIM-3 immunohistochemistry expression for patients with adenocarcinoma based on (A) DCR per RECIST v1.1 and (B) irDCR per irRECIST, and for patients with squamous cell histology based on (C) DCR per RECIST v1.1 and (D) irDCR per irRECIST

Supplementary Figure S5 Pretreatment PD-L1 expression for patients with adenocarcinoma based on (A) DCR per RECIST v1.1 and (B) irDCR per irRECIST, and for patients with squamous cell histology based on (C) DCR per RECIST v1.1 and (D) irDCR per irRECIST

Supplementary Table S1 Representativeness of study patients

Supplementary Table S2 Prior anticancer therapy regimens and associated response for the patient with an unconfirmed complete response

Supplementary Table S3 Tumor response for the patient with an unconfirmed complete response

## References

[1] Dong H , Zhu G, Tamada K, Chen L. B7-H1, a third member of the B7 family, co-stimulates T-cell proliferation and interleukin-10 secretion. Nat Med 1999;5:1365–9. 10581077 10.1038/70932

[2] Freeman GJ , Long AJ, Iwai Y, Bourque K, Chernova T, Nishimura H, . Engagement of the PD-1 immunoinhibitory receptor by a novel B7 family member leads to negative regulation of lymphocyte activation. J Exp Med 2000;192:1027–34. 11015443 10.1084/jem.192.7.1027 PMC2193311

[3] Latchman Y , Wood CR, Chernova T, Chaudhary D, Borde M, Chernova I, . PD-L2 is a second ligand for PD-1 and inhibits T cell activation. Nat Immunol 2001;2:261–8. 11224527 10.1038/85330

[4] Tseng SY , Otsuji M, Gorski K, Huang X, Slansky JE, Pai SI, . B7-DC, a new dendritic cell molecule with potent costimulatory properties for T cells. J Exp Med 2001;193:839–46. 11283156 10.1084/jem.193.7.839 PMC2193370

[5] Ribas A , Shin DS, Zaretsky J, Frederiksen J, Cornish A, Avramis E, . PD–1 blockade expands intratumoral memory T cells. Cancer Immunol Res 2016;4:194–203. 26787823 10.1158/2326-6066.CIR-15-0210 PMC4775381

[6] Fourcade J , Kudela P, Sun Z, Shen H, Land SR, Lenzner D, . PD-1 is a regulator of NY-ESO-1-specific CD8^+^ T cell expansion in melanoma patients. J Immunol 2009;182:5240–9. 19380770 10.4049/jimmunol.0803245 PMC3426222

[7] Iwai Y , Terawaki S, Honjo T. PD-1 blockade inhibits hematogenous spread of poorly immunogenic tumor cells by enhanced recruitment of effector T cells. Int Immunol 2005;17:133–44. 15611321 10.1093/intimm/dxh194

[8] Gandhi L , Rodríguez-Abreu D, Gadgeel S, Esteban E, Felip E, De Angelis F, . Pembrolizumab plus chemotherapy in metastatic non–small-cell lung cancer. N Engl J Med 2018;378:2078–92. 29658856 10.1056/NEJMoa1801005

[9] Gogishvili M , Melkadze T, Makharadze T, Giorgadze D, Dvorkin M, Penkov K, . Cemiplimab plus chemotherapy versus chemotherapy alone in non-small cell lung cancer: a randomized, controlled, double-blind phase 3 trial. Nat Med 2022;28:2374–80. 36008722 10.1038/s41591-022-01977-y PMC9671806

[10] Hellmann MD , Paz-Ares L, Bernabe Caro R, Zurawski B, Kim S-W, Carcereny Costa E, . Nivolumab plus ipilimumab in advanced non–small-cell lung cancer. N Engl J Med 2019;381:2020–31. 31562796 10.1056/NEJMoa1910231

[11] Johnson ML , Cho BC, Luft A, Alatorre-Alexander J, Geater SL, Laktionov K, . Durvalumab with or without tremelimumab in combination with chemotherapy as first-line therapy for metastatic non-small-cell lung cancer: the phase III POSEIDON study. J Clin Oncol 2023;41:1213–27. 36327426 10.1200/JCO.22.00975 PMC9937097

[12] Langer CJ , Gadgeel SM, Borghaei H, Papadimitrakopoulou VA, Patnaik A, Powell SF, . Carboplatin and pemetrexed with or without pembrolizumab for advanced, non-squamous non-small-cell lung cancer: a randomised, phase 2 cohort of the open-label KEYNOTE-021 study. Lancet Oncol 2016;17:1497–508. 27745820 10.1016/S1470-2045(16)30498-3 PMC6886237

[13] Paz-Ares L , Luft A, Vicente D, Tafreshi A, Gümüş M, Mazières J, . Pembrolizumab plus chemotherapy for squamous non–small-cell lung cancer. N Engl J Med 2018;379:2040–51. 30280635 10.1056/NEJMoa1810865

[14] Socinski MA , Jotte RM, Cappuzzo F, Orlandi F, Stroyakovskiy D, Nogami N, . Atezolizumab for first-line treatment of metastatic nonsquamous NSCLC. N Engl J Med 2018;378:2288–301. 29863955 10.1056/NEJMoa1716948

[15] Robert C , Schachter J, Long GV, Arance A, Grob JJ, Mortier L, . Pembrolizumab versus ipilimumab in advanced melanoma. N Engl J Med 2015;372:2521–32. 25891173 10.1056/NEJMoa1503093

[16] Motzer RJ , Escudier B, McDermott DF, George S, Hammers HJ, Srinivas S, . Nivolumab versus everolimus in advanced renal-cell carcinoma. N Engl J Med 2015;373:1803–13. 26406148 10.1056/NEJMoa1510665 PMC5719487

[17] Sui H , Ma N, Wang Y, Li H, Liu X, Su Y, . Anti-PD-1/PD-L1 therapy for non-small-cell lung cancer: toward personalized medicine and combination strategies. J Immunol Res 2018;2018:6984948. 30159341 10.1155/2018/6984948 PMC6109480

[18] American Cancer Society . Cancer facts & figures 2023. Atlanta: American Cancer Society; 2023 [cited 2023 Sep 11]. Available from: https://www.cancer.org/content/dam/cancer-org/research/cancer-facts-and-statistics/annual-cancer-facts-and-figures/2023/2023-cancer-facts-and-figures.pdf.

[19] Roque K , Ruiz R, Mas L, Pozza DH, Vancini M, Silva Júnior JA, . Update in Immunotherapy for advanced non-small cell lung cancer: optimizing treatment sequencing and identifying the best choices. Cancers 2023;15:4547. 37760516 10.3390/cancers15184547 PMC10526179

[20] Lim SM , Peters S, Ortega Granados AL, Pinto GDJ, Fuentes CS, Lo Russo G, . Dostarlimab or pembrolizumab plus chemotherapy in previously untreated metastatic non-squamous non-small cell lung cancer: the randomized PERLA phase II trial. Nat Commun 2023;14:7301. 37951954 10.1038/s41467-023-42900-4 PMC10640551

[21] Fourcade J , Sun Z, Pagliano O, Chauvin JM, Sander C, Janjic B, . PD-1 and Tim-3 regulate the expansion of tumor antigen-specific CD8^+^ T cells induced by melanoma vaccines. Cancer Res 2014;74:1045–55. 24343228 10.1158/0008-5472.CAN-13-2908 PMC3952491

[22] Jin HT , Anderson AC, Tan WG, West EE, Ha SJ, Araki K, . Cooperation of Tim-3 and PD-1 in CD8 T-cell exhaustion during chronic viral infection. Proc Natl Acad Sci U S A 2010;107:14733–8. 20679213 10.1073/pnas.1009731107 PMC2930455

[23] Dixon KO , Tabaka M, Schramm MA, Xiao S, Tang R, Dionne D, . TIM-3 restrains anti-tumour immunity by regulating inflammasome activation. Nature 2021;595:101–6. 34108686 10.1038/s41586-021-03626-9 PMC8627694

[24] de Mingo Pulido Á , Hänggi K, Celias DP, Gardner A, Li J, Batista-Bittencourt B, . The inhibitory receptor TIM-3 limits activation of the cGAS-STING pathway in intra-tumoral dendritic cells by suppressing extracellular DNA uptake. Immunity 2021;54:1154–67.e7. 33979578 10.1016/j.immuni.2021.04.019 PMC8192496

[25] de Mingo Pulido Á , Gardner A, Hiebler S, Soliman H, Rugo HS, Krummel MF, . TIM-3 regulates CD103^+^ dendritic cell function and response to chemotherapy in breast cancer. Cancer Cell 2018;33:60–74.e6. 29316433 10.1016/j.ccell.2017.11.019 PMC5764109

[26] Zhang C , Xu L, Ma Y, Huang Y, Zhou L, Le H, . Increased TIM-3 expression in tumor-associated macrophages predicts a poorer prognosis in non-small cell lung cancer: a retrospective cohort study. J Thorac Dis 2023;15:1433–44. 37065598 10.21037/jtd-23-227 PMC10089863

[27] Zhuang X , Zhang X, Xia X, Zhang C, Liang X, Gao L, . Ectopic expression of TIM-3 in lung cancers: a potential independent prognostic factor for patients with NSCLC. Am J Clin Pathol 2012;137:978–85. 22586058 10.1309/AJCP9Q6OVLVSHTMY

[28] Datar I , Sanmamed MF, Wang J, Henick BS, Choi J, Badri T, . Expression analysis and significance of PD-1, LAG-3, and TIM-3 in human non–small cell lung cancer using spatially resolved and multiparametric single-cell analysis. Clin Cancer Res 2019;25:4663–73. 31053602 10.1158/1078-0432.CCR-18-4142 PMC7444693

[29] Sun F , Guo ZS, Gregory AD, Shapiro SD, Xiao G, Qu Z. Dual but not single PD-1 or TIM-3 blockade enhances oncolytic virotherapy in refractory lung cancer. J Immunother Cancer 2020;8:e000294. 32461344 10.1136/jitc-2019-000294 PMC7254155

[30] Fourcade J , Sun Z, Benallaoua M, Guillaume P, Luescher IF, Sander C, . Upregulation of Tim-3 and PD-1 expression is associated with tumor antigen–specific CD8^+^ T cell dysfunction in melanoma patients. J Exp Med 2010;207:2175–86. 20819923 10.1084/jem.20100637 PMC2947081

[31] Murtaza A , Laken H, Da Silva Correia J, McNeeley P, Altobell L, Zhang J, . Discovery of TSR-022, a novel, potent anti-human TIM-3 therapeutic antibody. Eur J Cancer 2016;69:S102.

[32] Falchook GS , Ribas A, Davar D, Eroglu Z, Wang JS, Luke JJ, . Phase 1 trial of TIM-3 inhibitor cobolimab monotherapy and in combination with PD-1 inhibitors nivolumab or dostarlimab (AMBER). J Clin Oncol 2022;40(Suppl):2504.

[33] Eisenhauer EA , Therasse P, Bogaerts J, Schwartz LH, Sargent D, Ford R, . New response evaluation criteria in solid tumours: revised RECIST guideline (version 1.1). Eur J Cancer 2009;45:228–47. 19097774 10.1016/j.ejca.2008.10.026

[34] Simon R . Optimal two-stage designs for phase II clinical trials. Control Clin Trials 1989;10:1–10. 2702835 10.1016/0197-2456(89)90015-9

[35] Brookmeyer R , Crowley JJ. A confidence interval for the median survival time. Biometrics 1982;38:29.

[36] Ribas A , Eroglu Z, Trigo Perez JMM, Di Pace B, Wang T, Ghosh S, . AMBER parts 1c and 1e: A phase 1 study of cobolimab plus dostarlimab in patients (pts) with advanced/metastatic melanoma. J Clin Oncol 2022;40(Suppl):Abstract 9513.

[37] Harding JJ , Moreno V, Bang YJ, Hong MH, Patnaik A, Trigo J, . Blocking TIM-3 in treatment-refractory advanced solid tumors: a phase ia/b study of LY3321367 with or without an anti-PD-L1 antibody. Clin Cancer Res 2021;27:2168–78. 33514524 10.1158/1078-0432.CCR-20-4405

[38] Curigliano G , Gelderblom H, Mach N, Doi T, Tai D, Forde PM, . Phase I/ib clinical trial of sabatolimab, an anti-TIM-3 antibody, alone and in combination with spartalizumab, an anti-PD-1 antibody, in advanced solid tumors. Clin Cancer Res 2021;27:3620–9. 33883177 10.1158/1078-0432.CCR-20-4746

[39] Zeidan AM , Ando K, Rauzy O, Turgut M, Wang MC, Cairoli R, . Sabatolimab plus hypomethylating agents in previously untreated patients with higher-risk myelodysplastic syndromes (STIMULUS-MDS1): a randomised, double-blind, placebo-controlled, phase 2 trial. Lancet Haematol 2024;11:e38–50. 38065203 10.1016/S2352-3026(23)00333-2

[40] McEachern K , Sharma G, Ghosh S, Ramaswamy S, Jenkins D. The antitumor efficacy of TIM-3 blockade in a murine model of sarcoma. J Immunother Cancer 2018;6:874–5.

[41] Acoba JD , Rho Y, Fukaya E. Phase II study of cobolimab in combination with dostarlimab for the treatment of advanced hepatocellular carcinoma. J Clin Oncol 2023;41(Suppl):580.

[42] Das M , Zhu C, Kuchroo VK. Tim-3 and its role in regulating anti-tumor immunity. Immunological Rev 2017;276:97–111. 10.1111/imr.12520 PMC5512889 28258697

[43] Chiba S , Baghdadi M, Akiba H, Yoshiyama H, Kinoshita I, Dosaka-Akita H, . Tumor-infiltrating DCs suppress nucleic acid-mediated innate immune responses through interactions between the receptor TIM-3 and the alarmin HMGB1. Nat Immunol 2012;13:832–42. 22842346 10.1038/ni.2376 PMC3622453

[44] Ruffell B , . Therapeautic targeting of intratumoral dendritic cells. Presented at: 2022 International Cancer Immunotherapy Conference. September 29, 2022.

[45] Davar D , Eroglu Z, Milhem M, Becerra C, Gutierrez M, Ribas A, . 596 AMBER, Part 2B: a phase 1 study of cobolimab plus dostarlimab in patients with advanced/metastatic non-small cell lung cancer (NSCLC) previously treated with anti-PD(L)-1 therapy. J Immunother Cancer 2023;11(Suppl 1):A678–A.

