# Supplementary material for: Combined Targeting of PD-1 and TIM-3 in Patients with Locally Advanced or Metastatic Non–Small Cell Lung Cancer: AMBER Part 2B
Source: Clin Cancer Res. 2025 Jun 24;31(16):3443–51. doi: 10.1158/1078-0432.CCR-25-0806 (PMC12351275; doi:10.1158/1078-0432.CCR-25-0806)
Supplement: Supplementary Methods S1 — Additional inclusion/exclusion criteria [file ccr-25-0806_supplementary_methods_s1_suppms1.docx]

**Supplementary Text**

**Inclusion criteria**

Patients were included in AMBER Part 2B if they were ≥18 years of age, had histologically proven locally advanced (unresectable) or metastatic NSCLC, had progressed following treatment with anti–PD-1/PD-(L)1 antibody. If the patient had a known EGFR mutation, they must have received an EGFR tyrosine kinase inhibitor (e.g., erlotinib, gefitinib, afatinib, osimertinib, or experimental). Similarly, if they had a known ALK translocation, they must have received an ALK inhibitor (e.g., crizotinib, ceritinib, alectinib, or experimental). Patients with a known ROS1 mutation must have received a ROS1 inhibitor (e.g., crizotinib). Patients also had an ECOG performance status of 0 or 1 and had adequate organ function, defined as:

- ANC ≥1500/μL
- Platelets ≥100,000/μL
- Hemoglobin ≥9 g/dL or ≥5.6 mmol/L
- Serum creatinine ≤1.5× upper limit of normal (ULN) or calculated creatinine clearance rate ≥50 mL/min using Cockcroft–Gault equation for patients with creatinine levels >1.5×institutional ULN
- International normalized ratio or prothrombin time ≤1.5×ULN unless patient is receiving anticoagulant therapy, as long as prothrombin time (PT) or partial thromboplastin time (PTT) is within therapeutic range of intended use of anticoagulants. Activated PTT ≤1.5×ULN unless patient is receiving anticoagulant therapy as long as PT or PTT is within therapeutic range of intended use of anticoagulants
- Albumin ≥3.0 g/dL

Patients in Part 2B were also required to have a fresh tumor tissue biopsy prior to dosing; archival tissue could also be provided if available. For the archival sample, tumor tissue must be requested from offsite locations and confirmed available prior to dosing. If a patient has had a biopsy prior to entering the 21-day screening period and within approximately 12 weeks of study treatment, that biopsy may be accepted as the baseline fresh biopsy.

All patients are also required to have lesions amenable to biopsy and to agree to tumor biopsies prior to the initiation of treatment (as noted above) approximately 4–6 weeks after initiating treatment, and, if possible, upon treatment discontinuation (for patients with progressive disease).

For patients with NSCLC with radiological confirmation that the tumor cannot be safely accessed, an exception to above requirements may be granted only after discussion with the Medical Monitor. Patients who otherwise are unwilling to consent to undergo biopsies would not be eligible to participate.

Female patients of childbearing potential must have a negative serum or urine pregnancy test within 72 hours prior to the date of the first dose of study medication or be of nonchildbearing potential. Non-childbearing potential is defined as:

- ≥45 years of age and has not had menses for >1 year.
- Amenorrhoeic for <2 years without a hysterectomy and oophorectomy and a follicle stimulating hormone value in the postmenopausal range upon pre-study (screening) evaluation.
- Post hysterectomy, bilateral oophorectomy or tubal ligation. Documented hysterectomy or oophorectomy must be confirmed with medical records of the actual procedure or confirmed by an ultrasound. Tubal ligation must be confirmed with medical records of the actual procedure, otherwise women of childbearing potential must have a negative pregnancy test at screening and at every visit.

Female patients of childbearing potential must agree to use a highly effective method of contraception with their partner starting with the Screening Visit through 150 days after the last dose of study therapy.

**Exclusion criteria**

Patients were excluded from AMBER Part 2B if they had prior treatment with anti–PD-1, anti–PD-L1, or anti–PD-L2 agent (that resulted in permanent discontinuation due to an AE) or an anti–LAG-3 or anti–TIM-3. Patients were also excluded if they had a history of Grade ≥3 irAE with prior immunotherapy, with the exception of non-clinically significant lab abnormalities.

Patients were also excluded if they had known uncontrolled central nervous system metastases and/or carcinomatous meningitis. Patients with previously treated brain metastases may participate provided they are stable (without evidence of progression by imaging for ≥4 weeks prior to the first dose of study treatment and any neurologic symptoms have returned to baseline), have no evidence of new or enlarging brain metastases, and are clinically stable off steroids for at least 7 days prior to study treatment. Carcinomatous meningitis precludes a patient from study participation regardless of clinical stability.

Patients with a known additional malignancy that progressed or required active treatment within the last 2 years were also excluded. Patients with a prior or concurrent malignancy whose natural history or treatment does not have the potential to interfere with the safety or efficacy assessment of the investigational regimen may be included only after discussion with the Medical Monitor.

Patients were also excluded if they were considered a poor medical risk due to a serious, uncontrolled medical disorder, nonmalignant systemic disease or active infection requiring systemic therapy. Specific examples include, but are not limited to, active, non-infectious pneumonitis, uncontrolled chronic obstructive pulmonary disease, uncontrolled ventricular arrhythmia, recent (within 90 days) myocardial infarction, uncontrolled major seizure disorder, unstable spinal cord compression, superior vena cava syndrome, or any psychiatric or substance abuse disorders that would interfere with cooperation with the requirements of the study (including obtaining informed consent).

If a patient was pregnant or breastfeeding or expecting to conceive children within the projected duration of the study, starting with the screening visit through 150 days, they were excluded from AMBER 2B.

Receipt of systemic steroid therapy or any other form of immunosuppressive therapy within 7 days prior to the first dose of study treatment or a diagnosis of immunodeficiency or active autoimmune disease that requires systemic treatment (i.e., with use of disease-modifying agents, corticosteroids or immunosuppressive drugs) excluded patients from the study. Replacement therapy (e.g., thyroxine, insulin, or physiologic corticosteroid replacement therapy up to 5 mg prednisone or equivalent for adrenal or pituitary insufficiency, etc.) is not considered a form of systemic treatment. Use of inhaled steroids, topical steroids, local injection of steroids, and steroid eye drops are allowed.

Patients were also excluded if they had a known history of human immunodeficiency virus infection or antibodies, known active hepatitis B (e.g., hepatitis B surface antigen [HBsAg] reactive) or hepatitis C (e.g., hepatitis C virus ribonucleic acid [HCV RNA] [qualitative] is detected), or a history of pneumonitis.

If the patient had not recovered (i.e., to Grade ≤1 or to baseline) from radiation- and chemotherapy-induced AEs, had received transfusion of blood products (including platelets or red blood cells) or had received administration of colony-stimulating factors (including G-CSF, granulocyte-macrophage colony-stimulating factor or recombinant erythropoietin) within 3 weeks prior to the first dose of study drug or had not recovered adequately (Grade ≤1) from AEs and/or complications from any major surgery prior to starting therapy, they were excluded.

Patients were also excluded from Part 2B if they were participating and receiving study therapy at the time of enrollment or had participated in a study of an investigational agent and received investigational therapy or used an investigational device within 4 weeks prior to the first dose of study drug. Receipt of prior anticancer therapy (chemotherapy, targeted therapies, radiotherapy, or immunotherapy) within 21 days, or less than 5 times the half-life of the most recent therapy prior to study Day 1, whichever is shorter, also excluded patients from participation. Palliative radiation therapy to a small field ≥1 week prior to Day 1 of study treatment may be allowed. Finally, patients were excluded if they had a known hypersensitivity to TSR-022 or TSR-042 components or excipients (see IB or drug label for details).
