# Supplementary material for: Combined Targeting of PD-1 and TIM-3 in Patients with Locally Advanced or Metastatic Non–Small Cell Lung Cancer: AMBER Part 2B
Source: Clin Cancer Res. 2025 Jun 24;31(16):3443–51. doi: 10.1158/1078-0432.CCR-25-0806 (PMC12351275; doi:10.1158/1078-0432.CCR-25-0806)
Supplement: Supplementary Figure S1 — AMBER Part 2B design [file ccr-25-0806_supplementary_figure_s1_suppfs1.docx]

**Supplementary Figure S1. AMBER Part 2B design**


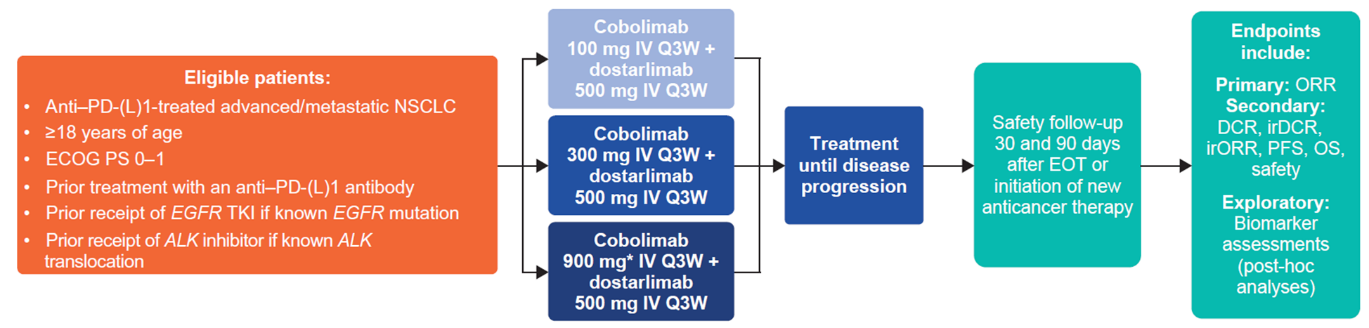
*Dose escalation into the 900 mg cohort in AMBER Part 2B was opened when the Safety Cohort in AMBER Part 1c dose escalation at 900 mg was cleared for safety.

*ALK*, anaplastic lymphoma kinase; DCR, disease control rate; ECOG PS, Eastern Cooperative Oncology Group performance status; *EGFR*, epidermal growth factor receptor; EOT, end of treatment; ir, immune-related; IV, intravenous; NSCLC, non-small cell lung cancer; ORR, overall response rate; OS, overall survival; PD-(L)1, programmed death ligand 1; PFS, progression-free survival; Q3W, every 3 weeks; RECIST (v1.1), Response Evaluation Criteria for Solid Tumors (version 1.1); TKI, tyrosine kinase inhibitor
