# Supplementary material for: Combined Targeting of PD-1 and TIM-3 in Patients with Locally Advanced or Metastatic Non–Small Cell Lung Cancer: AMBER Part 2B
Source: Clin Cancer Res. 2025 Jun 24;31(16):3443–51. doi: 10.1158/1078-0432.CCR-25-0806 (PMC12351275; doi:10.1158/1078-0432.CCR-25-0806)
Supplement: Supplementary Figure S2 — Post hoc analysis of prior ICI treatment and current exposure on study treatment [file ccr-25-0806_supplementary_figure_s2_suppfs2.docx]

**Supplementary Figure S2. Post hoc analysis of prior ICI treatment and current exposure on study treatment (N=70)^a,b^**  **
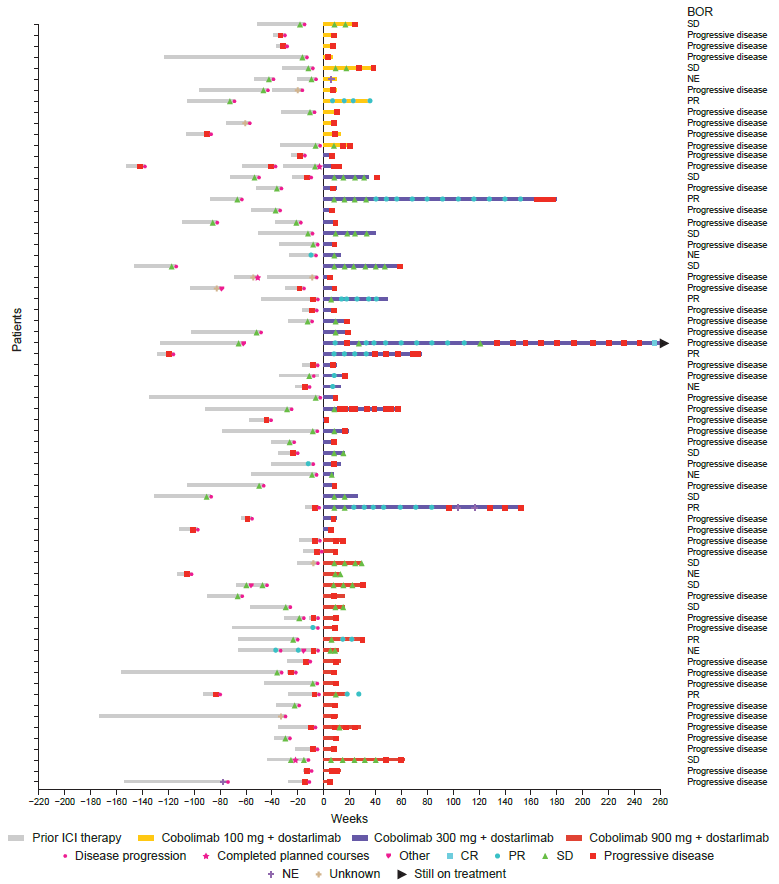
**^a^N number is defined as the safety population patient minus 14 patients whose BOR was categorized as ‘not done’; ^b^one subject received 'investigational drug' as prior anticancer treatment and this treatment was not included as a prior ICI. Confirmed BOR to study treatment is derived per RECIST v1.1. ‘Not done’ is defined as patients in the safety population who have no post-baseline tumor assessments. Overall responses are included if they occur on treatment or contribute to the confirmed BOR.

BOR, best overall response; CR, complete response; ICI, immune checkpoint inhibitor; NE, not evaluable; PR, partial response; RECIST v1.1, Response Evaluation Criteria for Solid Tumors version 1.1; SD, stable disease.
