# Supplementary material for: Combined Targeting of PD-1 and TIM-3 in Patients with Locally Advanced or Metastatic Non–Small Cell Lung Cancer: AMBER Part 2B
Source: Clin Cancer Res. 2025 Jun 24;31(16):3443–51. doi: 10.1158/1078-0432.CCR-25-0806 (PMC12351275; doi:10.1158/1078-0432.CCR-25-0806)
Supplement: Supplementary Figure S3 — Representative positron emission tomography (PET) and computed tomography (CT) images for the patient with an unconfirmed complete response [file ccr-25-0806_supplementary_figure_s3_suppfs3.docx]

**Supplementary Figure S3. Representative positron emission tomography (PET) and computed tomography (CT) images for the patient with an unconfirmed complete response**

**
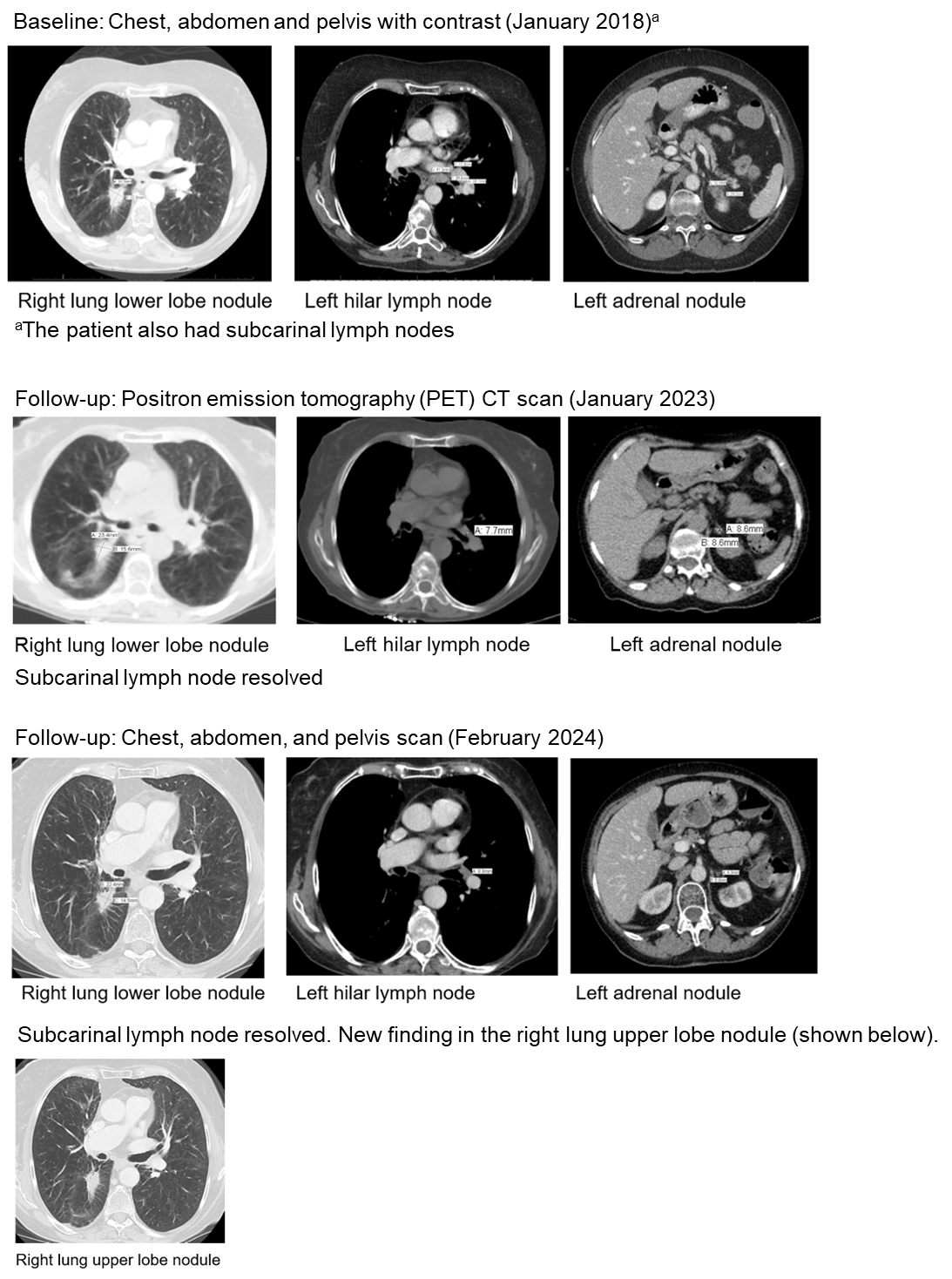
**
