# Supplementary material for: Combined Targeting of PD-1 and TIM-3 in Patients with Locally Advanced or Metastatic Non–Small Cell Lung Cancer: AMBER Part 2B
Source: Clin Cancer Res. 2025 Jun 24;31(16):3443–51. doi: 10.1158/1078-0432.CCR-25-0806 (PMC12351275; doi:10.1158/1078-0432.CCR-25-0806)
Supplement: Supplementary Figure S4 — Pretreatment TIM-3 immunohistochemistry expression for patients with adenocarcinoma based on (A) DCR per RECIST v1.1 and (B) irDCR per irRECIST, and for patients with squamous cell histology based on (C) DCR per RECIST v1.1 and (D) irDCR per irRECIST [file ccr-25-0806_supplementary_figure_s4_suppfs4.docx]

**Supplementary Figure S4. Pretreatment TIM-3 immunohistochemistry expression for patients with adenocarcinoma based on (A) DCR per RECIST v1.1 and (B) irDCR per irRECIST, and for patients with squamous cell histology based on (C) DCR per RECIST v1.1 and (D) irDCR per irRECIST**


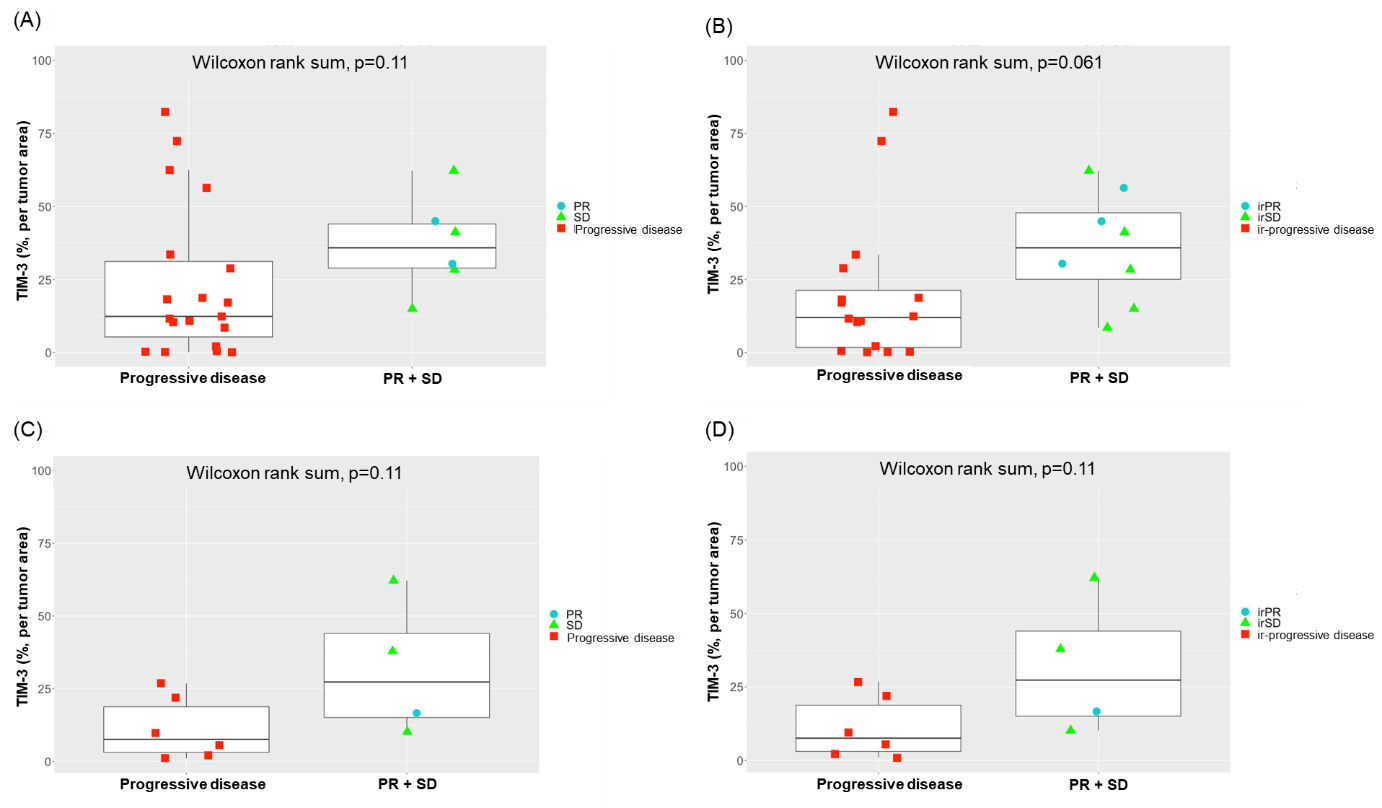
DCR, disease control rate; ir, immune-related; PR, partial response; RECIST v1.1, Response Evaluation Criteria in Solid Tumors version 1.1; SD, stable disease; TIM-3, T-cell immunoglobulin and mucin-domain-containing protein-3.
