# Supplementary material for: Combined Targeting of PD-1 and TIM-3 in Patients with Locally Advanced or Metastatic Non–Small Cell Lung Cancer: AMBER Part 2B
Source: Clin Cancer Res. 2025 Jun 24;31(16):3443–51. doi: 10.1158/1078-0432.CCR-25-0806 (PMC12351275; doi:10.1158/1078-0432.CCR-25-0806)
Supplement: Supplementary Table S1 — Representativeness of study patients [file ccr-25-0806_supplementary_table_s1_suppts1.docx]

**Supplementary Table S1. Representativeness of study patients**

| **Cancer type(s)/ subtype(s)/ stage(s)/ condition** | NSCLC |
| --- | --- |
| **Conditions related to:** | |
| **Sex** | As of 2020, NSCLC is reported to be slightly more common in men versus women, with men accounting for approximately 54–63% of cases (1).  In the United States, lung cancer incidence is rising sharply for women and particularly for younger and middle-aged women (2). |
| **Age** | NSCLC is typically found in older patient populations, with the median age at diagnosis of 70 years, with over a third of patients being 75 years or older (1). |
| **Race/ethnicity** | Although there are few studies that report survival outcomes by race (1), SEER data reports that approximately 75% of patients with NSCLC in the United States are White, 12% are Black, and 6% Hispanic (3).  Among men, Black patients have the highest incidence across all stages. Among women, White patients have the highest incidence; however, regional/distance stage NSCLC is highest among Black women (3).  2-year CSS is reported to be the highest in Hispanic patients regardless of gender, whereas Black men and women have a lower 2-year cause-specific survival rate than White men and women (3). Furthermore, people of color are more likely to present with a more advanced stage of disease at diagnosis than their White counterparts (3). |
| **Geography** | Across North America, Europe, and Asia, lung cancer is one of the most commonly diagnosed cancers and is associated with a high mortality rate versus other cancer (4). Mortality rates in Europe are higher than the global average and the 5-year survival rate is below 15% (4). |
| **Other considerations** | Prevalence of genetic alterations in oncogenic drivers is shown to vary across different regions, genders, ethnicity, and smoking history (4). |
| **Overall representativeness in this study** | The median age (67.5 years) and sex distribution of patients (41% female) in this study are in line with that reported in the literature.  The AMBER study recruited patients from the United States of America, Spain, Korea, and Taiwan. The majority of patients (85%) were White.  Genetic information was collected and this included genetic information for oncogenic drivers. |
| CSS, cause specific survival; NSCLC, non-small cell lung cancer; SEER, Surveillance, Epidemiology, and End Results.  1. Casal-Mouriño A, Ruano-Ravina A, Lorenzo-González M, Rodríguez-Martínez Á, Giraldo-Osorio A, Varela-Lema L*, et al.* Epidemiology of stage III lung cancer: frequency, diagnostic characteristics, and survival. *Transl Lung Cancer Res* 2021;**10**(1):506 doi 10.21037/tlcr.2020.03.40.  2. Jemal A, Schafer EJ, Sung H, Bandi P, Kratzer T, Islami F*, et al.* The burden of lung cancer in women compared with men in the US. *JAMA Oncol* 2023;**9**(12):1727-8 doi 10.1001/jamaoncol.2023.4415.  3. Primm KM, Zhao H, Hernandez DC, Chang S. Racial and ethnic trends and disparities in NSCLC. *JTO Clin Res Rep* 2022;**3**(8):100374 doi 10.1016/j.jtocrr.2022.100374.  4. Laguna JC, García-Pardo M, Alessi J, Barrios C, Singh N, Al-Shamsi HO*, et al.* Geographic differences in lung cancer: focus on carcinogens, genetic predisposition, and molecular epidemiology. *Ther Adv Med Oncol* 2024;**16**:17588359241231260 doi 10.1177/17588359241231260. | |
