# Supplementary material for: Combined Targeting of PD-1 and TIM-3 in Patients with Locally Advanced or Metastatic Non–Small Cell Lung Cancer: AMBER Part 2B
Source: Clin Cancer Res. 2025 Jun 24;31(16):3443–51. doi: 10.1158/1078-0432.CCR-25-0806 (PMC12351275; doi:10.1158/1078-0432.CCR-25-0806)
Supplement: Supplementary Table S2 — Prior anticancer therapy regimens and associated response for the patient with an unconfirmed complete response [file ccr-25-0806_supplementary_table_s2_suppts2.docx]

**Supplementary Table S2. Prior anticancer therapy regimens and associated response for the patient with an unconfirmed complete response**

| **Agent name  (regimen number)** | **Start and stop date** | **Best response** | **Date of progression or recurrence** |
| --- | --- | --- | --- |
| Cisplatin (1) | July 2012, 2012 | Unknown | October 2015 |
| Pemetrexed disodium (1) | July 2012,  March 2014 | Unknown | October 2015 |
| Erlotinib hydrochloride (2) | May 2014, September 2014 | Unknown | October 2015 |
| Nivolumab (3) | September 2015, November 2016 | Stable disease | May 2017 |
| Trametinib (4) | January 2017, January 2018 | Unknown | January 2018 |
| Dabrafenib (4) | July 2017, January 2018 | Unknown | January 2018 |
