# Supplementary material for: Combined Targeting of PD-1 and TIM-3 in Patients with Locally Advanced or Metastatic Non–Small Cell Lung Cancer: AMBER Part 2B
Source: Clin Cancer Res. 2025 Jun 24;31(16):3443–51. doi: 10.1158/1078-0432.CCR-25-0806 (PMC12351275; doi:10.1158/1078-0432.CCR-25-0806)
Supplement: Supplementary Table S3 — Tumor response for the patient with an unconfirmed complete response [file ccr-25-0806_supplementary_table_s3_suppts3.docx]

**Supplementary Table S3. Tumor response for the patient with an unconfirmed complete response**

|  | **24 Jan**  **2018 (Screening)** | **18 Apr 2018 (Day 64)** | **20 Jun 2018 (Day 127)** | **22 Aug 2018 (Day 190)** | **03 Oct 2018 (Day 232)** | **16 Mar**  **2020 (Day 762)** | **08 Jun 2020 (Day 846)** | **08 Sep 2020 (Day 938)** | **09 Jan 2023 (Day 1791)** |
| --- | --- | --- | --- | --- | --- | --- | --- | --- | --- |
| **Tumor measurement** | | | | | | | | | |
| **Target lesions** | | | | | | | | | |
| Lung (T01) | 30 mm (NA) | 25 mm (decreased) | 30 mm (increased) | 27 mm (decreased) | 17 mm (decreased) | 19 mm (stable) | 21 mm (increased) | 26.4 mm (increased) | 0 mm (absent) |
| Lymph node (T02) | 18 mm (NA) | 11 mm (decreased) | 17 mm (increased) | 10 mm (decreased) | 8 mm (decreased) | 10 mm (stable) | 10 mm (stable) | 10.4 mm (increased) | 0 mm (absent) |
| Adrenal gland (T03) | 26 mm (NA) | 16 mm (decreased) | 16 mm (stable) | 11 mm (decreased) | 9 mm (decreased) | 0 mm (absent) | 0 mm (absent) | 0 mm (Absent) | 0 mm (absent) |
| Sum of lesions | 74 mm | 52 mm | 63 mm | 48 mm | 34 mm | 29 mm | 31 mm | 36.8 mm | 0 mm |
| **Non-target lesion** | | | | | | | | | |
| Lymph node (NT01) | Present | Decreased | Increased | Decreased | Stable | Stable | Stable | Absent | Absent |
| **Overall response** | | | | | | | | | |
| **RECIST** | NA | Partial response | Progressive disease | Stable disease | Partial response | Partial response | Stable disease | Progressive disease | Complete response |
| **irRECIST** | NA | Partial response | Progressive disease | Stable disease | Partial response | Partial response | Stable disease | Progressive disease | Complete response |

irRECIST, immune-related Response Evaluation Criteria in Solid Tumors; NA, not applicable; RECIST, Response Evaluation Criteria in Solid Tumors.
